# Investigation of Thermal, Mechanical and Shape Memory Properties of 3D-Printed Functionally Graded Nanocomposite Materials †

**DOI:** 10.3390/nano13192658

**Published:** 2023-09-28

**Authors:** Mohamad Alsaadi, Eoin P. Hinchy, Conor T. McCarthy, Vicente F. Moritz, Alexandre Portela, Declan M. Devine

**Affiliations:** 1 CONFIRM Centre for Smart Manufacturing, University of Limerick, V94 T9PX Limerick, Ireland; eoin.hinchy@ul.ie (E.P.H.); conor.mccarthy@ul.ie (C.T.M.); 2 PRISM Research Institute, Technological University of the Shannon, Dublin Rd, N37 HD68 Athlone, Irelandalexandre.portela@ait.ie (A.P.); 3 Materials Engineering Department, University of Technology, Baghdad 10066, Iraq; 4 School of Engineering, University of Limerick, V94 T9PX Limerick, Ireland

**Keywords:** 4D printing, SLA, graphene nanoplatelets functionalisation, mechanical characteristics, fracture toughness, shape memory

## Abstract

In this study, a 3D-printed photocurable resin was developed by incorporating graphene nanoplatelets functionalised with melamine to investigate the thermal, mechanical, fracture and shape memory behaviours. The objective of this work was to produce a printed functionally graded nanocomposite material that has a smart temperature-responsive structure; presents good thermal stability, strength and fracture toughness; and can demonstrate shape-changing motions, such as sequential transformations, over time. The functionalised graphene nanoplatelets were examined via thermogravimetric analysis, Fourier transform infrared spectroscopy, Raman spectroscopy and ultraviolet–visible spectroscopy. Thermogravimetric analysis showed that the degradation temperature of the nanocomposite containing 0.1 wt% of functionalised graphene nanoplatelets at the weight loss of 5% was 304 °C, greater than that of the neat one by 29%. Dynamic mechanical analysis results showed property enhancements of the storage modulus and glass transition temperature. Fracture toughness, tensile strength and impact resistance were improved by 18%, 35% and 78%, respectively. The shape memory tests were performed to obtain the temperature-time recovery behaviour of the 3D-printed structures. The addition of functionalised graphene nanoplatelets demonstrated an enhancement in the shape recovery ratios. Generally, the five subsequent cycles were notably stable with a high recovery ratio of 97–100% for the flat shape and circular shape of the M-GNP specimens. On the other hand, these values were between 91% and 94% for the corresponding neat specimens.

## 1. Introduction

Given the fast technological development of additive manufacturing (AM) techniques, also called 3D printing, their employment in producing complex and high-precision 3D components for various applications has been explored by researchers. Hence, AM fabrication methods have been considered novel, leading manufacturing technologies [1,2]. One of the most widely used AM techniques for manufacturing prototypes, patterns and parts is stereolithography (SLA) 3D printing. In this technique, an ultraviolet (UV) laser beam is focused on a liquid photoreactive resin to crosslink the polymer chains, enabling the resin to cure layer-by-layer [3,4]. These resins mainly consist of liquid monomers/oligomers, crosslinkers and photoinitiators that are usually polymerised via a radical reaction process. SLA has attracted substantial attention due to its wide selection of materials, high-dimensional accuracy and quality surface finishes [5]. Nevertheless, the use of this technique for manufacturing advanced functionally graded materials with high mechanical and thermal properties still needs more development [6,7]. Therefore, several researchers have investigated the effect of employing fillers (nanoparticle and fibre) within the liquid-based resins on these properties [8,9]. 

Clear V4^TM^ is a standard urethane dimethacrylate-based photocurable FormLabs^TM^ resin used in conjunction with SLA 3D printing, which has attracted several studies for being a rigid material claimed to be suitable for designing injection-moulding inserts, high-resolution microfluidics structures, rapid prototyping, functional testing and product development [10,11,12,13]. Therefore, the 3D-printed Clear V4^TM^ resin has been used to improve its tensile strength and impact toughness through blending with copper nanoparticles [13]. Also, Inverardi et al. [14] investigated its sequential motion and shape memory properties with broad glass transition temperatures before and after post-curing.

The low interfacial adhesion between the polymer matrix and the reinforcement owing to nonhomogeneous dispersion and nanoparticle aggregations causes a stress concentration weakness [15]. Therefore, photopolymerisation techniques require improving the nanoparticle dispersion when using particulate-filled resins [6]. Several surface modification techniques, such as noncovalent or covalent functionalisation, have been employed to enhance nanoparticle dispersion and handle the agglomeration phenomena [16,17,18,19]. For instance, Uysal incorporated graphene oxide (GO) nanoparticles with contents of 0.05 to 0.25 wt% into an epoxy/acrylate-based SLA resin; parts were 3D-printed (3DP) with a UV light source of 405 nm and displayed improved tensile strength. Morphological investigations indicated that GO was homogeneously dispersed, without agglomeration, when using 3-(methacryloyloxy) propyl trimethoxysilane as a surface modifier [19].

Furthermore, carbon-based nanoparticles have been proven effective for 3D printing of photocurable resins, considerably enhancing the final properties by incorporating small nanoparticle concentrations. For example, Mu et al. [20] used digital light processing (DLP) 3D printing to study the tensile properties of a photocurable acrylic-based resin reinforced with 0.1 to 0.6 wt% multi-walled carbon nanotubes (MWCNT). The results demonstrated that, compared to the unmodified resin, the tensile modulus and strength of the nanocomposite exhibited improvement by 45% and 21%, respectively. A similar investigation by Santos et al. [21], who used 0.25 to 0.75 wt% MWCNT incorporated to epoxy/acrylate resin, resulted in enhanced tensile modulus and hardness by 42% and 77%, respectively, while the glass transition temperature (*T_g_*) increased by 10 °C. Compared to other nanoparticles, graphene nanoplatelets (GNP) have a unique size and platelet morphology and are made of a stacked-graphene-layers platelet morphology, which is widely used as an additive to improve the mechanical properties of lightweight composites in order to obtain functional and structural nanocomposites [22,23,24]. Chowdhury [25] manufactured 4DP components of graphene nanoparticles modified by acrylate-based shape memory nanocomposites using a Micro-SLA 3D printer. It was reported that the inclusion of 0.1 and 0.3 wt% graphene nanoparticles slightly increased the tensile strength and *T_g_*. The author concluded that employing the SLA technique for graphene nanocomposite resins can create new prospects for designing novel shape memory components. 

Shape memory polymers have been acknowledged as a unique group of smart materials, and, in recent years, 4DP shape memory polymers and their composites (SMPs/SMPCs) have been used as functionally graded materials due to the unique properties of their lightweight structure, ease of processing and low cost [26]. The dynamic, smart structure of 4DP polymers can be activated via an external stimulus, prompting the shape and morphological transitions that could be effectively monitored and correspond to macroscopic movements [27]. A number of distinct mechanisms may be employed to achieve the desired shape-change response, but thermoresponsive approaches, such as in as thermally triggered SMP materials, are the most studied. The mechanism of thermally triggered SMPs is defined as a (Permanent) recovery shape that can be set as (Temporary) through a thermomechanical process to obtain a desired programming shape [14,28,29]. The functionally graded materials and structures have attracted significant attention as materials whose structures and compositions have the capability to switch shape, thereby enabling the optimisation of the properties for desired applications [30]. Idowu et al. [31] manufactured 3DP GNP/epoxy nanocomposites utilising direct ink writing (DIW) to investigate the mechanical, thermal and shape memory properties. Results indicated that a GNP addition of 0.1 wt% displayed 15% faster shape recovery owing to enhanced thermal conductivity, evidence of a strong interface between GNP and the polymeric matrix. The modulus and tensile strength were also increased by 17% and 30%, respectively. Furthermore, the loss tangent and storage modulus were considerably enhanced with 66% and 365%, respectively, compared to unmodified 3DP specimens, demonstrating improved damping characteristics. The authors concluded that this novel resin would be a potential candidate for self-assembling structures and energy-efficient actuation systems.

As indicated by the above literature, the thermal, mechanical, fracture toughness and shape memory behaviours of various nanoparticles reinforced with 3DP composites have been investigated. However, these studies have not explored the effects of functionalised GNP using melamine (M-GNP) on these characteristics of the 3DP components. In this work, an SLA 3D-printed photocurable resin was employed to fabricate M-GNP nanocomposites. The functionalisation of GNP was examined via Fourier transform infrared spectroscopy (FTIR), thermogravimetric analysis (TGA), Raman spectroscopy and ultraviolet-visible (UV-Vis) spectroscopy. Thermal stability was characterised by TGA, and thermomechanical behaviour was evaluated by dynamic mechanical analysis (DMA). The mechanical and fracture characteristics were investigated by tensile, Izod impact and single-edge notch bending (SENB) tests. The fracture surface morphology of the tensile specimens was then observed via scanning electron microscopy (SEM).

The final goal was to assess the viability of 3D-printing a commercial material and turning it into a functionally graded nanocomposite material to obtain and exploit high-performance 4DP structures without synthesising a complex material. In other words, this study aimed at manufacturing active components in order to gain a new path for novel applications, e.g., in robotics as a compliant mechanism and passive thermal sensor or in aerospace as a self-deployable structure.

## 2. Materials and Methods

### 2.1. Materials

Clear V4^TM^ resin (referred to as neat resin) (FormLabs^TM^ Inc., Somerville, MA, USA) was employed as the photocurable resin with a mixture of 55–75% urethane dimethacrylate (UDMA), 15–25% methacrylate monomers and <0.09% diphenyl (2,4,6 trimethylbenzoyl) phosphine oxide (BAPO) as photoinitiator. The GNP was employed with a thickness of 15 nm and particle size of 5 μm, bulk density of 0.03–0.1 g·cm^−3^ and surface area of 50–80 m^2^·g^−1^. Melamine (2,4,6-Triamino-1,3,5-triazine, sym-Triaminotriazine) with a purity of 99% was employed for GNP functionalising and N,N-dimethylformamide (DMF) to dissolve GNP and melamine. All materials were provided by Sigma-Aldrich (Dorset, UK).

### 2.2. M-GNP Preparation

**Figure 1:**
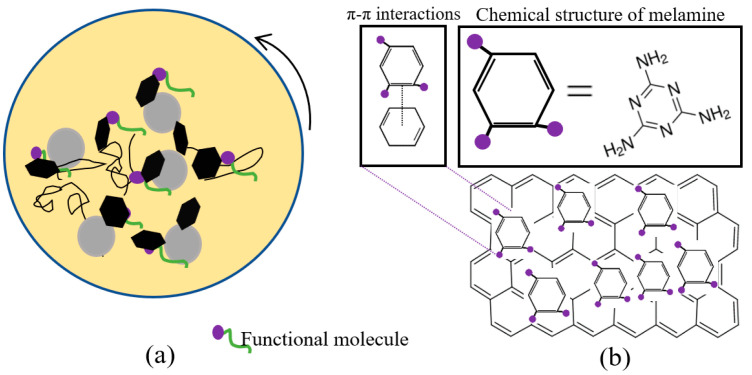
Schematic diagram of the functionalisation process of GNP: (**a**) Ball-milling and functional molecules; (**b**) The GNP functionalisation by melamine.

A total of 250 mg of melamine was dissolved and stirred in 75 mL of DMF for 15 min. Then, the blend was ultrasonicated after adding 250 mg of GNP for 15 min. To functionalise GNP, the mixture was ball-milled for 24 h (Figure 1a). The hexagonal rings of melamine were stuck on the GNP surface to generate π–π interactions (Figure 1b). Then, the blend was vacuum-filtered for 45 min. Finally, the M-GNP was vacuum dried at room temperature.

### 2.3. Nanocomposite Resin Preparation

The neat resin was mixed with M-GNP at 40 °C to decrease the resin viscosity and enhance the dispersion of the M-GNP. Formulations were prepared by adding contents of 0.03, 0.06 and 0.1 wt% of functionalised M-GNP. The mixture was blended at 1500 rpm for 30 min in a planetary centrifugal mixer (Thinky Mixer ARM-310 CE). Finally, the resin mixture was mixed at 200 rpm under a 0.1 bar vacuum for 30 min to remove the air bubbles. The 3D printing process was conducted by adding the prepared resin to the vat of a Form Lab 2 SLA 3D printer.

### 2.4. 3D Printing Process

The 3DP specimens were manufactured using a Form Lab 2 SLA 3D printer (FormLabs^TM^ Inc., Somerville, MA, USA) with 0.05 mm layer thickness and 405 nm wavelength. The support structure was oriented to the printed specimens relative to the platform with an angle of 45° to provide a good quality and resolution for the printed structure. After removing the support structure, the 3DP specimens were washed in two steps for 30 s in each step by isopropyl alcohol (IPA). The 3DP specimens were post-cured in two stages for 60 min in each stage: first under UV light of 1.25 mW·cm^−2^ and then in an oven at 60 °C as recommended by the resin manufacturer.

### 2.5. Characterisation Process

#### 2.5.1. Characterisation of the Functionalised Graphene Nanoplatelets

The modified M-GNP compound was characterised by FTIR (ATR-FTIR, Perkin Elmer Spectrum Inc., Norwalk, CT, USA)), TGA (Pyris 1 TGA, Perkin Elmer), Raman spectroscopy (Renishaw Invia, New Mills, UK) and UV-Vis spectroscopy (UV-1280, Shimadzu Europa, Duisburg, Germany). FTIR was used to investigate the chemical compositions. TGA was employed to obtain the thermal decomposition behaviour of the M-GNP, melamine and pristine GNP powders at the temperature zones of 25 °C to 1000 °C with a rising range of 10 °C·min^−1^ in a nitrogen atmosphere. Raman spectroscopy was performed to present the shapeshifting of M-GNP using a laser (Green 532 nm, 50× objective lens and Grating 1800 lines·mm^−1^). The dispersion of the M-GNP was observed using UV-Vis spectroscopy.

#### 2.5.2. Characterisation of Thermal, Mechanical and Fracture Toughness of the 3DP Specimens

TGA was employed to investigate the thermal stability and mass decomposition of the 3DP specimens with the same procedure used in the previous section but with a final temperature of 600 °C. DMA was used to investigate the dynamic thermomechanical behaviour of the 3DP specimens using a PerkinElmer machine (Waltham, MA, USA). A single cantilever mode with an oscillation frequency of 1 Hz and strain of 0.02% was used at temperature zones of 30 °C to 160 °C and a heating range of 3 °C·min^−1^, with specimen dimensions of 17.5 × 12.0 × 3.2 mm^3^. *T_g_* was determined as the *tan(δ)* peak temperature.

The tensile test was conducted using a Zwick Roell machine (Z010, GmbH & Co. KG, DEU, Sundern, Germany) with a crosshead speed of 1 mm·min^−1^ and a loadcell capacity of 10 kN in accordance with ASTM D638 type V specimens, with a gauge length of 7.62 mm. In order to evaluate the dispersion quality, the fracture surfaces of the failed specimens were investigated using Mira SEM (Tescan, Oxford Instruments, Cambridge, UK). The Izod impact test was carried out on an Izod impact machine (Instron CEAST) with a 5.5 J hammer according to the ASTM D4812 standard for unnotched specimens. At least three specimens were experimented for each test.

The fracture toughness behaviour was investigated using a Zwick Roell machine by calculating the stress intensity factor of the SENB specimens according to the ASTM standard D5045-99. The pre-crack was introduced in the 3D printing process. The crosshead speed was 2 mm·min^−1^. The SENB specimens were printed with a size of 52.80 × 12.00 × 6.35 mm^3^. The crack length (*a*) was selected such that the $\frac{a}{W}$ ratio was approximately 0.45 (W is the specimen width). The SENB specimens were examined to determine the critical stress intensity factor (*K_A_*) (also called plain strain fracture toughness); hence, it is a toughness parameter indicative of the resistance of a material to fracture. The opening mode I fracture toughness was considered, and according to the linear elastic fracture mechanics (LEFM) theory, *K_A_* values were calculated from the following equations (ASTM D5045-99):

(1) $$K_{A}=\left(\frac{P}{{BW}^{\frac{1}{2}}}\right)f\left(\frac{a}{W}\right)$$

(2) $$f\left(\frac{a}{W}\right)=\frac{3sA^{1/2}}{2W}\frac{1.99-A\left(1-A\right)\left(2.15-3.93A+2.7A^{2}\right)}{\left(1+2A\right){\left(1-A\right)}^{\frac{3}{2}}}$$

where $A=\frac{a}{W}$, *P* is the fracture load, *B* is the specimen thickness and $f\left(\frac{a}{W}\right)$ is the correction parameter.

#### 2.5.3. Characterisation of the Temperature-Memory Effect of the 3DP Specimens

**Figure 2:**
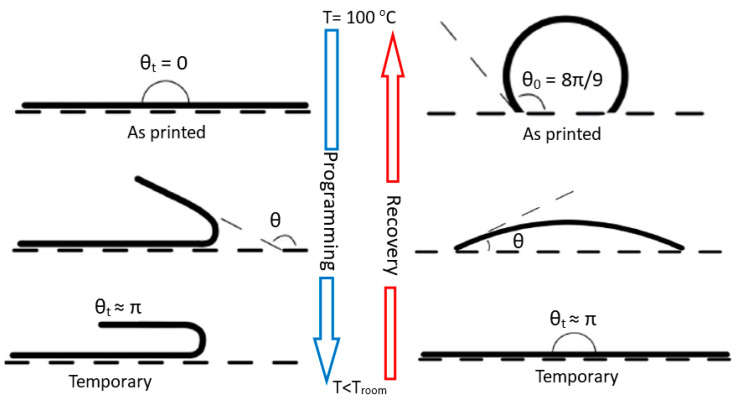
Schematic diagram outlining the programming and recovery stages for the 3DP flat-shaped specimen and the circular-shaped specimen (angle *θ* is used to evaluate the shape recovery).

The temperature-memory effect was investigated for two types of 3DP geometries, namely the flat-shaped specimen and the circular-shaped specimen to perform a multiple, controlled and sequential deployment on a 3DP structure. Figure 2 represents the schematic illustration outlining the programming and recovery stages for the 3DP specimens. The flat-shaped specimen was printed with dimensions of 50.0 × 7.0 × 1.5 mm^3^, while the circular-shaped specimen was printed with an angle of 8π/9, radius of 7 mm and thickness of 1.5 mm. The shape memory behaviour was divided into two stages: the first was the temporary shape (programming) obtained after heating the specimen in an oven to a temperature close to the *T_g_* (100 °C), followed by the cooling process to reach a low temperature (0 °C) by putting the specimens in a fridge. The second stage, i.e., the permanent shape (recovery), was performed by heating (0 °C to 100 °C) in an oven (Figure 2).

The recovery process was recorded with a high-resolution camera placed in front of an oven with a transparent glass window and controlled to take four photos per minute. The temperature was measured with a thermocouple placed near the specimen. The changes in the shape, i.e., the strain recovery, were quantified by measuring the angle of the specimen (*θ*) (Figure 2) with digital imaging analysis software (ImageJ, version 1.54d), and the recovery ratio ($R_{a}$) was evaluated by the equation below:

(3) $$R_{a}\left(\%\right)=\frac{\theta -\theta_{t}}{\theta_{o}-\theta_{t}}\times 100\%$$

where *θ_t_* is the angle of the flat-shaped specimen in the temporary shape (*θ_t_* = π), *θ* is the actual angle and *θ_o_* is the original angle (*θ_o_* = 0). On the other hand, for the circular-shaped specimen, *θ_t_* is the angle in the temporary shape (*θ_t_* = 0), *θ* is the actual angle and *θ_o_* is the original angle (*θ_o_* = 8π/9). All angles were measured in radians.

## 3. Results and Discussion

### 3.1. Characterisation of the Functionalised GNP

**Figure 3:**
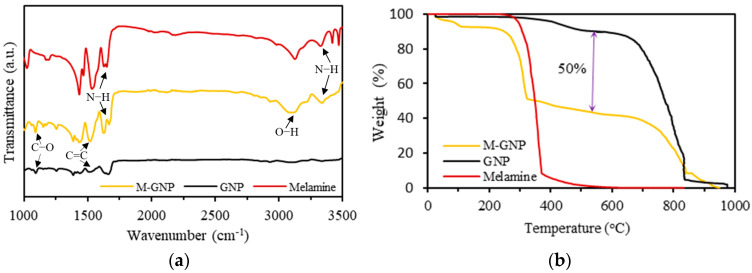
Characterisation of the M-GNP, GNP and melamine: (**a**) FTIR spectra; (**b**) TGA.

The noncovalent functionalisation of GNP was confirmed using spectra analysis. The FTIR of M-GNP, pristine GNP and melamine are presented in Figure 3a. The M-GNP and melamine spectra displayed remarkable signals at 1620 cm^−1^ and 3310 cm^−1^, which revealed the presence of N–H bonds. Additionally, peaks of C=C stretching and C–O stretching of the pristine GNP were located on the M-GNP at 1590 cm^−1^ and 1075 cm^−1^. These peaks prove that the GNP surface is functionalised through the low-energy ball milling procedure [32,33,34,35]. Moreover, a stretched broad peak appeared at about 3090 cm^−1^ as new O–H bonds in the M-GNP originated from the ball milling mechanochemical procedure [35].

The functionalisation of GNP was also verified using TGA (Figure 3b). The mass decomposition of M-GNP is assumed to be a combination of pristine GNP and melamine. M-GNP showed a high thermal degradation of melamine above 250 °C. Then, it was noticed to become similar to the carbonisation of GNP in the zone between 300 and 700 °C. Additionally, the mass loss of M-GNP was around 50% in this zone, as evidenced by the differences in the neat compounds’ thermal curves, demonstrating the functionalisation effectiveness.

**Figure 4:**
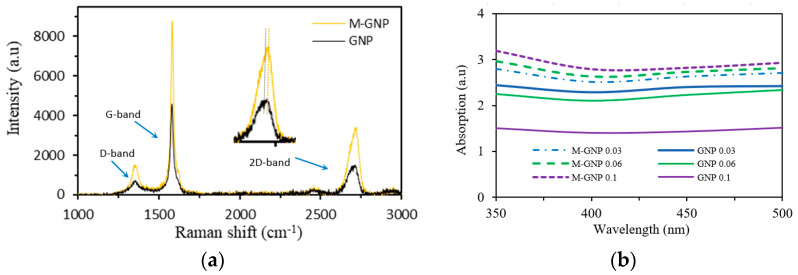
Characterisation of the M-GNP and GNP: (**a**) Shapeshifting via Raman spectra; (**b**) UV light absorption via UV-Vis spectra in acetone solution.

The Raman spectra are observed from 500 cm^−1^ to 3000 cm^−1^ for GNP and show D-, G- and 2D bands at 1344 cm^−1^, 1577 cm^−1^ and after 2701 cm^−1^ as presented in Figure 4a. The M-GNP had a similar form, since noncovalent interactions, i.e., a functionalisation process, cannot affect the intrinsic behaviour of GNP [36]. Nonetheless, there was a small shape-changing and -shifting in the 2D band of M-GNP, which was 2710 cm^−1^ (up-shifted 9 cm^−1^). The functionalisation process led to a change in the intensity values due to the few-layer graphene interacting with melamine because of π–π interactions. The measurements show a change in the intensity level, producing a small shift for the M-GNP 2D band. Therefore, this behaviour of M-GNP suggested that the GNP surface interacted with melamine through affinity by π–π interactions [35,36].

The quality of M-GNP and pristine GNP dispersion was observed and quantified by UV-Vis spectroscopy (Figure 4b). According to this method, the individual M-GNP in the acetone solution greatly absorb UV light at wavelengths between about 350 nm and 500 nm; on the other hand, agglomerated GNP in the acetone solution absorb less UV light since the stacked GNP blocks UV light. In addition, these agglomerated particles tend to precipitate in a heterogeneous solution. Thus, at a certain wavelength, the absorption of UV light can be associated with a measurement of the degree of dispersion [35,37,38,39]. In this test, the concentration selected was 1 mg·mL^−1^ for all acetone solutions, and the test was conducted after 1 h from the time of mixing. Since several individual layers of graphene were stacked, pristine GNP exhibited low absorption, as shown in Figure 4b, with the maximum absorbance recorded for the 0.1 wt% M-GNP solution. This result suggests the potential success of fabricating 3DP photosensitive resin nanocomposites with enhanced mechanical and thermal characteristics [35,40].

### 3.2. Thermal Behaviour of the 3DP Nanocomposites

The thermal degradation of the 3DP M-GNP nanocomposites was assessed via TGA. The degradation temperatures (*T*_5_*)* at the weight loss of 5% and the percentage of residual weight (*W*_400_*)* at the temperature of 400 °C are presented in Table 1. The results suggest that incorporating M-GNP considerably improved the thermal stability of the photopolymer matrix. *T*_5_ of the 0.1 wt% M-GNP nanocomposite was 304 °C, 29% greater than that of the neat resin. Similarly, *W*_400_ of the pure material was 56%, while for M-GNP 0.1, this value was 77%, an increment of 38%. The enhanced thermal stability with such a small amount of M-GNP was influenced by the good interfacial adhesion between the 3DP polymer matrix and M-GNP due to the functionalisation of melamine via π–π interactions and the improved dispersion of the GNP that improved the UV curing reaction of the photocurable 3DP resin [33].

**Figure 5:**
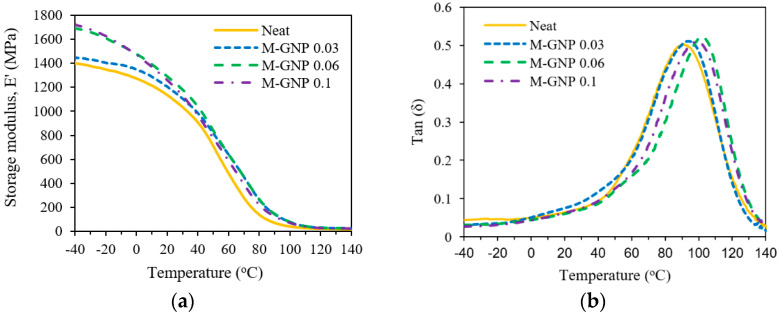
Dynamic mechanical analysis: (**a**) Storage modulus, *E’* and (**b**) damping factor (*tan(δ)*).

The conformation of macromolecular chains and the viscoelastic behaviours of polymers can be examined via DMA through exposure to a wide range of temperatures under an oscillating load. Hence, the change in *T_g_* can be attributed to the movement and mobility of the macromolecular chains. [41]. The storage modulus (*E*′) and the loss tangent (*tan(δ)*) of the 3DP neat material and the modified nanocomposites are presented in Figure 5a and 5b, respectively. It is observed that with the addition of M-GNP, all curves of *E*′ and *T_g_* are shifted to the right, and all the values are slightly greater than those of the neat resin. The macromolecule motion is mainly constrained by two factors: (i) Mechanical interlocking in the vicinity of a nanoparticle due to the roughness introduced by it; and (ii) the improved polymer-functionalised graphene interface, comprising physical interactions such as π–π, and residual functional groups on the M-GNP surfaces may interact with functional groups of the macromolecule [42,43]. The enhancement in *T_g_* with incorporating M-GNP suggests an increasing intensity level of interactions between the polymer matrix and M-GNP, which is endorsed by the improved thermal stability obtained by incorporating the functionalised graphene.

### 3.3. Mechanical and Fracture Toughness Behaviour of the 3DP Nanocomposites

The results of the tensile and impact tests of the 3DP nanocomposites are presented in Table 2. The tensile strength and impact resistance results rose gradually with increasing M-GNP content. For instance, the tensile strength and impact resistance of the 0.1 wt% M-GNP nanocomposite increased, respectively, by 35% and 78% compared to those of the neat material. The elastic modulus values were also enhanced with the addition of M-GNP relative to the pure photosensitive resin.

**Figure 6:**
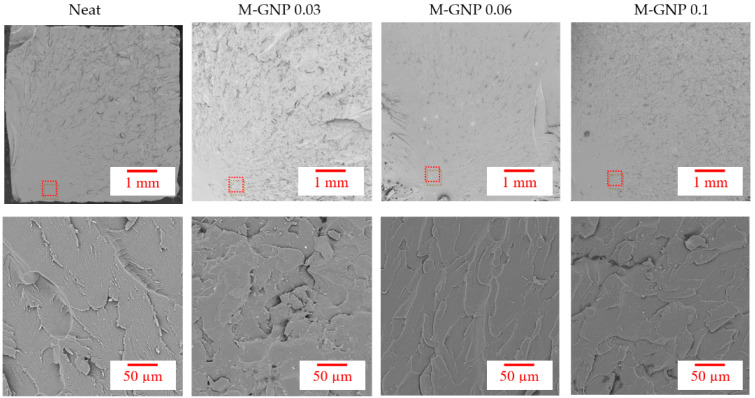
SEM micrographs of the tensile test specimen fracture surface (Red frame represents the location of the 50 µm image).

SEM surface morphology fractographies of the neat specimen and nanocomposites are displayed in Figure 6. Agglomerations of M-GNP were not visible within the resin matrix. The M-GNP nanoparticles were found to be embedded and homogeneously dispersed throughout the matrix, evincing that melamine prevented GNP particles from aggregating through π–π interactions between their surfaces. Furthermore, the amino groups (-NH_2_) of melamine likely bonded covalently with the polymer, leading to a strong affinity and interfacial interaction with the matrix system [34]. These interactions caused the load transfer to improve across the matrix–nanoparticle interface [34]. Thus, the multilayer M-GNP’s larger specific surface area promotes greater strength for the nanocomposites, endorsing the results obtained for the dynamic thermomechanical behaviour.

**Figure 7:**
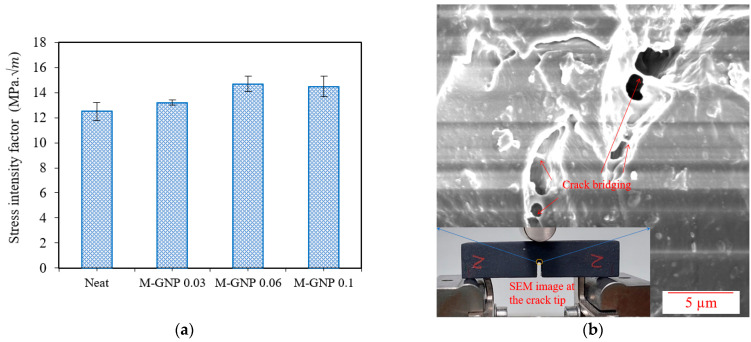
(**a**) Stress intensity factor values of the SENB test (**b**) SEM of 0.1% M-GNP SENB specimen.

As shown in Figure 7a, the *K_A_* values were improved gradually with the inclusion of M-GNP. The maximum enhancement was 17.6% compared to that of the neat resin, obtained at 0.06 wt% M-GNP. Hence, the strong interface bonding between M-GNP and the photosensitive resin led GNP nanoparticles to pin the crack front and offer additional crack growth resistance, demonstrating an improvement in the fracture toughness behaviour of the nanoparticulate polymer composites [44,45]. Furthermore, as presented in Figure 7b of the SEM micrograph for the interface between the neat matrix and M-GNP particles for the SENB specimen, incorporating M-GNP, the nanoparticle pull-out and crack bridging were the dominant toughening mechanisms. Hence, energy is dissipated by the frictional pull-out of the particles from the polymer matrix, which leads to bridging the crack, thus delaying crack opening and propagation [46].

### 3.4. Temperature-Memory Behaviour of the 3DP Nanocomposites

**Figure 8:**
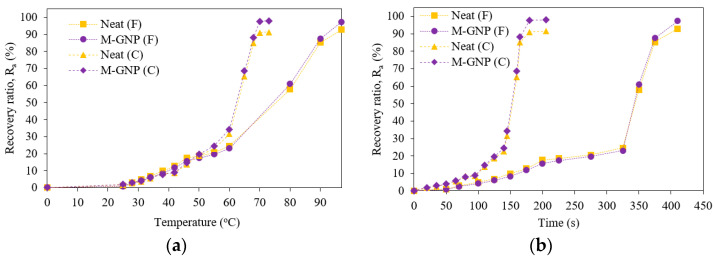
Shape memory behaviour. (**a**) Recovery ratio versus temperature and (**b**) Recovery ratio versus time.

**Figure 9:**
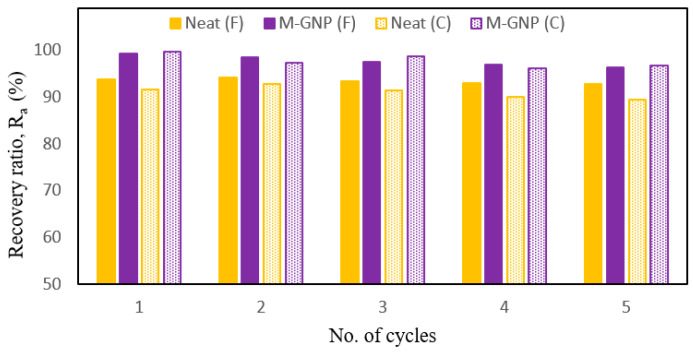
Shape recovery behaviour of the repeated thermomechanical cycles.

Figure 8a,b present the shape recovery ratio versus temperature and shape recovery ratio versus time, respectively, for the neat specimens and the M-GNP (0.1 wt%) specimens as average results of four repeated shape memory test cycles. It was noticed that both specimens gradually recovered their original shape from *T* = 0 °C up to a temperature of 73 °C for the circular-shaped (C) and 97 °C for the flat-shaped (F). This behaviour indicates that the shape recovery process could occur at the glassy-to-rubbery transition region. Hence, in the glassy region (below *T_g_*), the recovery procedure may include slow chain mobility attributed to the relaxation process. While close to and above *T_g_*, recovery happens due to a relaxation process based on entropic forces acting on chains that gain motion [14]. In addition, the difference in the *R_a_* values between the M-GNP specimens and neat specimens for the circular specimen is greater than that of the flat specimen. This behaviour is due to the incorporation of graphene within polymeric-based nanocomposites, which can promote quick switches from the programmed shape back to their original shape [25,31,47,48]. However, it is worthy of note that the conditions under which the test was carried out are far from the small-strain linear viscoelastic region; hence, this method is based on approximations [14]. This type of testing method is performed to assist in describing the response of the temperature-time recovery behaviour. Figure 9 shows the shape recovery behaviour of the repeated thermomechanical cycles. The average *R_a_* for the five thermomechanical cycles of the circular shape was 91.1% for the neat specimen and 97.8% for the M-GNP specimen. On the other hand, the average *R_a_* of the flat shape was 93.6% for the neat specimen and 97.7% for the M-GNP specimen. In other words, the five subsequent cycles were stable with a high recovery ratio range of 97–100% for the flat shape and circular shape of the M-GNP specimens. On the other hand, these values were between 91% and 94% for the corresponding neat specimens.

## 4. Conclusions

In this study, a dimethacrylate-based photocurable resin of Form Lab 2 printer was used to explore the effects of the noncovalent functionalisation of GNP with melamine on the thermal stability, storage modulus, glass transition temperature, tensile strength, impact fracture toughness and shape-memory behaviour of the 3DP specimens.

The GNP functionalisation was validated via TGA, FTIR, Raman spectroscopy and UV-VIS spectroscopy. The enhancement in thermal, mechanical and fracture toughness properties with low contents of M-GNP was due to the good interfacial adhesion between the functionalised GNP and the polymer matrix. This behaviour is attributed to the noncovalent melamine functionalisation promoted by π–π interactions, leading to a homogeneous GNP dispersion and an inhibition of surface defect creation, as observed by UV-VIS spectroscopy and SEM images of the failed tensile specimens, as well as leading to an enhancement of the UV photocuring reaction of the dimethacrylate-based resin.

The results demonstrated enhancements of up to 38% in residual weight at 400 °C, 35% in tensile strength and 78% in impact strength with the addition of M-GNP of 0.1 wt%. The maximum increment of the critical stress intensity factor and *T_g_* were 17.6% and 12 °C, respectively, obtained at M-GNP of 0.06 wt%. The values of the thermal degradation, storage modulus and glass transition temperature were improved with the inclusion of M-GNP. Shape memory tests were performed to evaluate the recovery ratios of the 3DP circular-shaped and flat-shaped specimens for the neat and 0.1 wt% of M-GNP. This testing method was applied to assist in describing the response of the temperature-time recovery behaviour. The addition of M-GNP showed improved shape recovery ratios and decreased the time to recover the original shape for both shapes of the specimens over five repeated specimens. The recovery ratio was in the range of 97–100% for the flat-shape and circular-shape M-GNP specimens, while the value was between 91% and 94% for the corresponding neat specimens.

## Figures and Tables

**Table 1:** Thermal degradation of the 3DP nanocomposites.

Material	Neat	M-GNP 0.03	M-GNP 0.06	M-GNP 0.1
*T*_5_ (°C)	236	288	296	304
*W*_400_ (%)	56	63	72	77

**Table 2:** Tensile and Izod impact properties of the 3D-printed nanocomposites.

Material Type	Tensile Strength (MPa)	Tensile Modulus (GPa)	Impact Resistance (kJ·m^−2^)
Neat	60.5 ± 0.6	2651 ± 87	6.5 ± 0.4
M-GNP 0.03	73.9 ± 0.7	2958 ± 96	9.2 ± 0.3
M-GNP 0.06	77.3 ± 3.4	2693 ± 105	10.5 ± 0.9
M-GNP 0.1	81.1 ± 4.1	2774 ± 134	11.6 ± 0.8

## Data Availability

The data presented in this study are available on request from the corresponding author.
